# Blood Glucose Screening Rates Among Minnesota Adults With Hypertension, Behavioral Risk Factor Surveillance System, 2011

**DOI:** 10.5888/pcd11.140204

**Published:** 2014-11-26

**Authors:** Renée S.M. Kidney, James M. Peacock, Steven A. Smith

**Affiliations:** Author Affiliations: James M. Peacock, Minnesota Department of Health, St Paul, Minnesota; Steven A. Smith, Mayo Clinic, Rochester, Minnesota. Dr Smith is also affiliated with the Mayo College of Medicine, Rochester, Minnesota.

## Abstract

**Introduction:**

Many US adults have multiple chronic conditions, and hypertension and diabetes are among the most common dyads. Diabetes and prediabetes prevalence are increasing, and both conditions negatively affect cardiovascular health. Early diagnosis and treatment of diabetes and prediabetes can benefit people with hypertension by preventing cardiovascular complications.

**Methods:**

We analyzed 2011 Minnesota Behavioral Risk Factor Surveillance System data to describe the proportion of adults with hypertension screened for diabetes according to US Preventive Services Task Force Recommendations for blood glucose testing. Covariates associated with lower odds of recent screening among adults without diabetes were determined using weighted logistic regression.

**Results:**

Of Minnesota adults with self-reported hypertension, 19.6% had a diagnosis of diabetes and 10.7% had a diagnosis of prediabetes. Nearly one-third of adults with hypertension without diabetes had not received blood glucose screening in the past 3 years. Factors associated with greater odds of not being screened in multivariable models included being aged 18 to 44 years (adjusted odds ratio [AOR], 1.77; 95% confidence interval [CI], 1.23–2.55); being nonobese, with stronger effects for normal body mass index; having no check-up in the past 2 years (AOR, 2.49; 95% CI, 1.49–4.17); having hypertension treated with medication (AOR, 2.01; 95% CI, 1.49–2.71); and completing less than a college degree (AOR, 1.45; 95% CI, 1.14–1.84). Excluding respondents with prediabetes or those not receiving a check-up did not change the results.

**Conclusions:**

Failure to screen among providers and failure to understand the importance of screening among individuals with hypertension may mean missed opportunities for early detection, clinical management, and prevention of diabetes.

## Introduction

In 2010, 26% of US adults lived with 2 or more chronic conditions (1), a number expected to increase as the prevalence of chronic conditions increases (2). The most common pairing of multiple chronic conditions across ages is diabetes and hypertension (1). In Minnesota, the adult diabetes prevalence nearly doubled in the past 15 years to more than 7%, and 26% of adults report receiving a hypertension diagnosis (3,4).

Recent evidence-based guidelines for prevention of diabetes and cardiovascular risk emphasize expanded delivery of preventive clinical services, which may improve referral to diabetes prevention programs and early clinical management for adults with diabetes (5,6). Blood pressure control among adults with diabetes significantly reduces cardiovascular events (7,8). On the basis of blood pressure data, the US Preventive Services Task Force (USPSTF) rated blood glucose screening for people with diagnosed hypertension a Grade B recommendation, making it a covered preventive service under the Affordable Care Act (9).

Despite the value of diabetes screening in patients with hypertension, estimates of the implementation of USPSTF screening guidelines are unavailable. National Health and Nutrition Examination Survey (NHANES) data from 2005–2006 indicate that 53.2% of adults with hypertension have been screened (10); recent analyses were limited to attributes of different screening criteria, including sensitivity, specificity, and absolute numbers of cases detected (11,12). Assessment of screening rates and patient-level characteristics associated with screening status are needed.

This analysis describes blood glucose screening rates and patient-level characteristics among Minnesota adults with hypertension. The results provide baseline information for developing public health and clinical strategies to identify patients with hypertension who are at risk for diabetes.

## Methods

To assess blood glucose screening rates, we obtained data for Minnesota from the 2011 Behavioral Risk Factor Surveillance System (BRFSS) (13), a state-based survey of health-related behaviors among noninstitutionalized US adults aged 18 years or older and administered by states for the Centers for Disease Control and Prevention (CDC). Standardized clinical measures assessing diabetes management do exist, but standardized clinical measures for assessing diabetes screening rates do not exist. BRFSS provides a unique opportunity to assess statewide trends.

We limited analysis to 2011 data because differences in sampling and weighting initiated for the 2011 survey make results noncomparable to those of previous years. In Minnesota, the percentage of people with complete or partially complete interviews (ie, cooperation rate), was 82.2%, and the completion rate was 51.9%, higher than the national medians of 73.8% (cooperation rate) and 49.7% (completion rate) (14). We identified a cross-sectional sample of adults (N = 3,847) with self-reported hypertension, available covariate data, self-reported diabetes or prediabetes status, and blood glucose screening in the previous 3 years.

We identified all people with hypertension and then determined subsets of people who would closely meet the definition of asymptomatic for diabetes used in the USPSTF recommendation (Figure). All responses of “don’t know/not sure” or “refused” were treated as missing.

**Figure:**
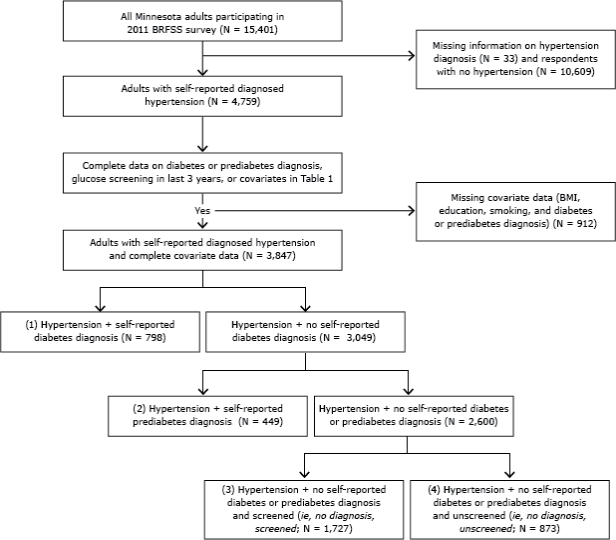
Flow diagram describing cohort with hypertension (N = 3,847), classified according to diabetes status, prediabetes status, and self-reported blood glucose screening for diabetes in the last 3 years, Minnesota Behavioral Risk Factor Surveillance System (BRFSS), 2011. Abbreviations: BMI, body mass index; screened, self-reported blood glucose screening.

### Diagnoses and screening

Respondents were asked a series of questions relating to high blood pressure and diabetes (15). All adults who responded yes to the question, “Have you ever been told by a doctor, nurse, or other health professional that you have high blood pressure?” were considered to have hypertension. Similar to the procedure used by Kilmer et al (16), we assigned a composite variable for diabetes, prediabetes, and screening. Diabetes status was ascertained by asking people whether a “doctor, nurse, or other health professional ever told you that you . . . have diabetes?” Women who responded yes were asked, “Was this only when you were pregnant?” If they had diabetes only when pregnant, they were considered not to have diabetes. For all other yes responses, the respondent was classified as having diabetes. Adults with hypertension not classified as having diabetes were asked, “Have you ever been told by a doctor or other health professional that you have prediabetes or borderline diabetes?” Women were asked “Was this only when you were pregnant?” If women responded yes, they were coded as not having prediabetes. All others who responded affirmatively were coded as having prediabetes. All adults with hypertension were classified as having self-reported diabetes, self-reported prediabetes, or neither.

To assess screening among adults with hypertension but no diabetes diagnosis, these adults were asked, “Have you had a test for high blood sugar or diabetes within the past 3 years?” Yes and no responses classified individuals as screened and not screened, respectively (15).

We used the diagnosis-screening variable to create 4 groups: 1) self-reported diabetes diagnosis; 2) self-reported prediabetes diagnosis; 3) no self-reported diabetes or prediabetes diagnosis and self-reported glucose screening in the past 3 years; 4) no self-reported diabetes or prediabetes diagnosis and no self-reported glucose screening in the past 3 years (Figure).

### Covariates

Covariates were included because they are risk factors for hypertension or diabetes (12), are associated with health literacy, may be associated with access to care, and are associated with patterns of medical care use and disease severity. Variables were age (18–44, 45–64, or ≥65 years), sex (male or female), race/ethnicity (non-Hispanic white or other), education (collapsed into college graduate or not), smoking (collapsed into current or not). Body mass index (BMI, calculated by using self-reported height and weight [kg/m^2^]) was classified as low/normal (<25.0), overweight (25.0–29.9) and obese (≥30.0). Respondents with hypertension were classified by whether they were taking medication to control their hypertension. Health insurance status over the last year was classified as yes or no. Check-up was defined as “a general physical exam, not an exam for a specific injury, illness, or condition” (15), and the responses were either yes or no. Two years was selected as the time frame for the last check-up, because it most closely aligns with the 3-year glucose screening interval.

### Analysis

Analyses were conducted by using SAS 9.2 (SAS Institute, Inc) and survey procedures to incorporate sampling weights into the analysis. Symmetric 95% confidence intervals (CIs) were calculated for frequencies. Differences between proportions were tested by using a Rao-Scott χ^2^ test. All results were weighted to adjust for nonresponse rates and probability of selection into the survey. Missing data were treated as not-missing-at-random to be more conservative with estimates of standard error.

Proc SurveyFreq was used to calculate frequencies for all adults with diagnosed hypertension and the subset who did not report diabetes. Additionally, frequencies were generated using procedures similar to those used by Kilmer et al (16) to understand relations between demographic, lifestyle, or health care access variables and the spectrum of glycemic states among adults with hypertension, because the proportion of adults with diagnosed diabetes also influences the proportion of adults with hypertension who were eligible for screening and other presented results. Sensitivity analyses were conducted to examine whether patterns were influenced by people with coronary heart disease (CHD). As Kilmer et al did (16), we removed adults with hypertension who had CHD according to survey questions about history of myocardial infarction, angina, and coronary heart disease. Differences in the distribution of diabetes and prediabetes diagnosis or screening across levels of each covariate were tested by using a Rao-Scott χ^2^ test.

We used logistic regression to determine associations (odds ratios [ORs]) between demographic, lifestyle, and health care access variables and blood glucose screening in the past 3 years. Univariate associations were determined for all covariates for the sample and all respondents with hypertension who had available data. Results did not differ. A multivariable model included all potential covariates to determine the effect of each covariate after controlling for all others in the model. A model including all significant covariates (*P* < .05*)* is presented. We tested models that included an interaction term between age and BMI and found the interaction term to be nonsignificant. Three sensitivity analyses were conducted: 1 model limiting analyses to respondents who had had a check-up within the past 2 years, on the assumption that this visit provided an opportunity for preventive efforts like blood glucose screening; 1 limited to non-Hispanic whites, because the small number of respondents from other racial/ethnic groups prevented analysis by race/ethnicity or adjustment; and 1 excluding respondents who had diagnosed prediabetes, because clinical guidelines recommend annual blood glucose monitoring (17), increasing the probability of screening. Among individuals with prediabetes, 90% reported having had a blood glucose test in the past 3 years.

## Results

Weighted frequencies were used to describe Minnesota adults with diagnosed hypertension (Table 1). Most were 45 years or older (81%) and non-Hispanic white (90%); there were slightly more men than women in this group. Nearly 80% were classified as either overweight or obese. Approximately 22% were college graduates. More than 90% had insurance coverage and had a check-up in the past 2 years. Less than 1 in 7 reported coronary heart disease, and 20% reported a diagnosis of diabetes. Characteristics were not appreciably different for the subset of individuals with hypertension who had no diabetes diagnosis (Table 1), and 67.6% of these adults reported blood glucose testing within the past 3 years.

**Table 1:** Demographics and Health-Related Conditions Among Minnesota Adults With Self-Reported Hypertension, Behavioral Risk Factor Surveillance System, 2011

Variable	Self-Reported Hypertension Diagnosis and Complete Covariates (N = 3,847)		Self-Reported Hypertension Diagnosis and Complete Covariates, Excluding Those with Diabetes Diagnosis (N = 3,049)	
	N	Weighted % (95% CI)	N	Weighted % (95% CI)
**Age, y**				
18–44	416	19.0 (16.8–21.2)	374	21.3 (18.7–23.9)
45–64	1,690	42.5 (40.3–44.7)	1,352	41.9 (39.4–44.4)
≥65	1,741	38.5 (36.4–40.6)	1,323	36.9 (34.5–39.2)
**Sex**				
Male	1,803	54.2 (51.9–56.4)	1,402	53.6 (51.1–56.1)
Female	2,044	45.8 (43.6–48.1)	1,647	46.4 (43.9–48.9)
**Body mass index category (kg/m^2^)**				
Low/normal (<25.0)	866	21.4 (19.6–23.2)	775	23.7 (21.6–25.8)
Overweight (25.0–29.9)	1,482	38.3 (36.2–40.5)	1,226	39.7 (37.3–42.2)
Obese (≥30)	1,499	40.3 (38.0–42.5)	1,048	36.5 (34.0–39.1)
**Education**				
Less than high school	244	11.9 (10.1–13.7)	168	10.7 (8.7–12.6)
High school graduate	1,210	33.5 (31.3–35.6)	924	32.8 (30.5–35.2)
Attend college/trade school	1,169	32.4 (30.3–34.6)	930	33.5 (31.0–35.9)
College graduate	1,224	22.3 (20.6–23.8)	1,027	23.0 (21.2–24.9)
**Race/ethnicity**				
Not-Hispanic white	3,538	89.8 (88.2–91.4)	2,838	90.7 (88.9–92.5)
Other	309	10.2 (8.5–11.8)	211	9.3 (7.5–11.1)
**Enrolled in a health plan**				
Yes	3,622	92.1 (90.5–93.7)	2,867	92.0 (90.3–93.8)
No	225	7.9 (6.3–9.5)	182	8.0 (6.2–9.7)
**Had a check-up in the past 2 years**				
Yes	3,617	92.1 (90.5–93.6)	2,852	91.7 (89.9–93.4)
No	230	7.9 (6.4–9.5)	197	8.3 (6.6–10.1)
**Smoking history**				
Current	562	17.8 (15.8–19.8)	461	19.1 (16.7–21.5)
Former	1,531	38.1 (36.0–40.3)	1,194	37.3 (34.8–39.7)
Never	1,754	44.0 (41.8–46.3)	1,394	43.6 (41.2–46.1)
**Ever had a heart attack**				
Yes	370	10.0 (8.6–11.4)	247	8.3 (6.8–9.7)
No	3,453	90.0 (88.6–91.4)	2,787	91.3 (89.8–92.8)
Missing	24	—a	15	—a
**Ever had angina or coronary heart disease diagnosis**				
Yes	383	9.2 (8.0–10.5)	246	7.3 (6.1–8.6)
No	3,431	90.8 (89.5–92.0)	2,784	92.0 (90.7–93.3)
Missing	33	—a	19	—a
**Total coronary heart disease diagnosis**				
Yes	566	14.6 (13.0–16.2)	377	12.2 (10.5–13.8)
No	3,251	85.4 (83.8–87.0)	2,650	87.1 (85.5–88.8)
Missing	30	—a	22	—a
**Taking hypertension medication**				
Yes	3,181	77.5 (75.4–79.7)	2,442	74.2 (71.6–76.7)
No	666	22.5 (20.3–24.6)	607	25.8 (23.3–28.4)
**Diagnosis**				
Diabetes diagnosis	798	19.6 (17.9–21.3)	0	0
Prediabetes diagnosis	449	10.7 (9.3–12.0)	449	13.3 (11.6–14.9)
Neither	2,600	69.7 (67.7–71.8)	2,600	86.7 (85.0–88.4)
**Blood glucose testing in past 3 years (among those without diabetes)**				
Yes	2,140	67.6 (65.1–70.0)	2,140	67.6 (65.1–70.0)
No	909	32.4 (30.0–34.9)	909	32.4 (30.0–34.9)
Excluded people with diabetes	798	NA		NA

Abbreviations: CI, confidence interval; NA, not applicable.

a A dash ( — ) indicates below reporting threshold of n = 50 needed to generate reliable frequencies.

Other descriptive analysis focused on 4 groups of people with hypertension: those diagnosed with diabetes (19.6%), those diagnosed with prediabetes (10.7%), those diagnosed with neither diabetes nor prediabetes and screened in the past 3 years (no diagnosis, screened, 44.6%), and those diagnosed with neither diabetes nor prediabetes but not screened in the past 3 years (no diagnosis, unscreened, 25.1%) (Table 2). We examined the proportion of adults in each composite diagnosis-screening group stratified by diabetes risk factors and access to the health care system (Table 2). The percentage of adults with diabetes tended to be higher among adults who were obese, had lower educational attainment, were of a race/ethnicity other than non-Hispanic white, and not current smokers. Compared with their counterparts, the percentage of adults who had no diagnosis and were unscreened was greater among adults who were younger, had lower BMI, were current smokers, were not enrolled in a health plan, and did not have a recent check-up. We also conducted analyses excluding the 14.6% of adults who had coronary heart disease; associations were unchanged.

**Table 2:** Blood Glucose Screening Results Among Adults With Hypertension in Minnesota, by Demographic, Lifestyle, and Health Care Access Variables, Behavioral Risk Factor Surveillance System, 2011

Variable	Diabetes		Prediabetes		Neither, Screened		Neither, Unscreened		*P* Value
	N	% (95% CI)	N	% (95% CI)	N	% (95% CI)	N	% (95% CI)	
**Overall**	798	19.6 (17.9–21.3)	449	10.7 (9.3–12.0)	1,727	44.6 (42.3–46.9)	873	25.1 (23.1–27.2)	NA
**Age, y**									
18–44	42	—a	30	—a	173	40.3 (33.6–47.1)	171	44.4 (37.5–51.3)	<.001
45–64	338	20.8 (18.1–23.5)	210	12.0 (9.8–14.3)	774	46.1 (42.8–49.4)	368	21.1 (18.5–23.7)	
≥65	418	23.0 (20.4–25.6)	209	11.8 (9.8–13.8)	780	45.1 (42.0–48.2)	334	20.1 (17.7–22.5)	
**Sex**									
Male	401	20.4 (17.9–23.0)	192	10.3 (8.2–12.3)	787	43.6 (40.3–46.9)	423	25.7 (22.8–28.6)	.57
Female	397	18.6 (16.3–20.9)	257	11.1 (9.4–12.9)	940	45.8 (42.8–48.8)	450	24.4 (21.6–27.3)	
**BMI category (kg/m^2^)**									
Low/normal (<25.0)	91	11.0 (8.1–13.8)	76	8.3 (5.7–10.8)	416	45.9 (41.1–50.7)	283	34.9 (30.3–39.4)	<.001
Overweight (25.0–29.9)	256	16.6 (14.0–19.3)	162	10.5 (8.3–12.6)	716	46.5 (43.0–50.1)	348	26.4 (23.1–29.6)	
Obese (≥30)	451	27.0 (24.0–30.1)	211	12.1 (9.8–14.4)	595	42.1 (38.4–45.8)	242	18.8 (15.5–22.0)	
**Education**									
Less than college graduate	601	20.5 (18.4–22.5)	297	10.5 (8.9–12.1)	1,107	43.0 (40.3–45.6)	618	26.1 (23.6–28.6)	.007
College graduate	197	16.6 (13.7–19.5)	152	11.3 (9.0–13.6)	620	50.4 (46.5–54.2)	255	21.7 (18.6–24.8)	
**Race/ethnicity**									
Non-Hispanic white	700	18.8 (17.1–20.5)	416	10.4 (9.1–11.7)	1,613	45.2 (42.9–47.5)	809	25.5 (23.4–27.7)	.10
Other	98	26.7 (19.7–33.7)	33	—a	114	39.0 (30.5–47.6)	64	21.4 (15.0–27.9)	
**Smoking status**									
Current	101	13.9 (10.2–17.6)	65	8.7 (5.3–12.0)	237	43.3 (36.8–49.8)	159	34.1 (27.6–40.7)	<.001
Former	337	21.4 (18.6–24.2)	210	13.6 (11.1–16.1)	667	43.2 (39.7–46.7)	317	21.8 (18.9–24.7)	
Never	360	20.3 (17.7–23.0)	174	9.0 (7.2–10.7)	823	46.3 (43.2–49.5)	397	24.4 (21.6–27.1)	
**Enrolled in health plan**									
Yes	755	19.7 (17.9–21.4)	433	11.1 (9.6–12.5)	1,645	45.1 (42.8–47.4)	789	24.1 (22.1–26.2)	.02
No	43	—a	16	—a	82	38.9 (28.2–49.6)	84	36.7 (26.9–46.4)	
**Had a check-up in the past 2 years**									
Yes	765	20.0 (18.2–21.7)	428	10.8 (9.4–12.1)	1,665	46.4 (44.1–48.7)	759	22.9 (20.9–24.9)	<.001
No	33	—a	21	—a	62	24.0 (15.1–32.9)	114	51.3 (40.8–61.7)	
**Use blood pressure medications**									
Yes	739	23.1 (21.1–25.1)	395	11.9 (10.3–13.4)	1,456	46.1 (43.7–48.5)	591	18.9 (17.1–20.7)	<.001
No	59	7.5 (5.1–9.9)	54	6.6 (3.7–9.4)	271	39.4 (33.9–44.9)	282	46.5 (40.8–52.3)	

Abbreviations: CI, confidence interval; BMI, body mass index.

a A dash ( — ) indicates below reporting threshold of n = 50 needed to generate reliable frequencies

To describe associations between demographic, lifestyle, and health care access and use variables and screening among respondents at risk of developing diabetes, we calculated the odds of not being tested within the past 3 years (Table 3). Adults aged 18 to 44 were 2.64 (95% CI, 1.89–3.67) times as likely to not be screened compared with adults aged 65 years or older; there was no meaningful difference for adults aged 45 to 64 versus those aged 65 years or older. Odds of not being screened were elevated for adults with low/normal BMI compared with those who were obese, for those without a college degree compared with those with a college degree, and for current smokers compared with never smokers. Adults not taking medication to manage their hypertension were less likely to be screened (OR, 2.94; 95% CI, 2.25–3.85) than those who did take medication. Nonenrollment in a health plan (OR, 1.97; 95% CI, 1.22–3.17) and lack of a check-up in the past 2 years (OR, 3.70; 95% CI, 2.28–6.02) were associated with not being screened. In multivariable models, most associations remained, with similar magnitude of effect. Smoking was no longer associated with screening, nor was health plan enrollment. Removing covariates that were significant at *P *< .05 did not appreciably change the overall results or point estimates of associations between covariates and no screening. None of the 3 sensitivity analyses changed results appreciably. For example, in data limited to non-Hispanic whites only, associations between respondents aged 18 to 44 years (OR, 1.90; 95% CI, 1.28–2.81) and not having a check-up (OR, 3.08; 95% CI, 1.93–4.91) were strengthened slightly. If limited to respondents without a prediabetes diagnosis, the association with not having a check-up was strengthened (OR, 3.10; 95% CI, 1.83–5.24).

**Table 3:** Odds of Not Having a Blood Glucose Test in the Last 3 Years Among Minnesota Adults with Hypertension and No Diabetes (N = 3,049), Behavioral Risk Factor Surveillance System, 2011

Variable	Unadjusted Associations (Univariable Model)		Adjusted Associations (Multivariable Model)^a^		Full Model^a^	
	OR (95% CI)	*P* Value	OR (95% CI)	*P* Value	OR (95% CI)	*P* Value
**Age, y**						
18–44	2.64 (1.89–3.67)	<.001	1.83 (1.26–2.67)	.002	1.77 (1.23–2.55)	.002
45–64	1.07 (0.85–1.34)	.57	1.01 (0.78–1.31)	.92	1.02 (0.79–1.30)	.91
≥65	1 [Reference]					
**Sex**						
Male	1.10 (0.90–1.39)	.36	1.10 (0.87–1.39)	.44	—	
Female	1 [Reference]				—	
**BMI category (kg/m^2^)**						
Low/normal (<25)	1.78 (1.32–2.39)	<.001	2.24 (1.64–3.06)	<.001	2.18 (1.60–2.96)	<.001
Overweight (25–29.9)	1.25 (0.95–1.64)	.12	1.43 (1.08–1.89)	.01	1.44 (1.08–1.90)	.01
Obese (>30)	1 [Reference]					
**Education**						
Less than college graduate	1.41 (1.12–1.77)	.004	1.50 (1.17–1.92)	.001	1.45 (1.14–1.84)	.003
College graduate	1 [Reference]					
**Race/ethnicity**						
Non-Hispanic white	1 [Reference]				—	
Other	0.99 (0.64–1.52)	.95	0.62 (0.37–1.05)	.08	—	
**Smoking history**						
Current	1.47 (1.05–2.07)	.03	0.83 (0.59–1.18)	.30	—	
Former	0.86 (0.68–1.08)	.20	0.84 (0.65–1.07)	.15	—	
Never	1 [Reference]				—	
**Enrolled in health plan**						
Yes	1 [Reference]				—	
No	1.97 (1.22–3.17)	.006	1.15 (0.67–1.96)	.62	—	
**Had a check-up in the last 2 Years**						
Yes	1 [Reference]					
No	3.70 (2.28–6.02)	<.001	2.56 (1.56–4.20)	<.001	2.49 (1.49–4.17)	<.001
**Taking hypertensive medication**						
Yes	1 [Reference]					
No	2.94 (2.25–3.85)	<.001	2.05 (1.52–2.78)	<.001	2.01 (1.49–2.71)	<.001

Abbreviations: OR, odds ratio; CI, confidence interval; —, variable was not included in model; BMI, body mass index.

a The multivariable model includes all covariates; the full model includes only variables that were significant at *P* < .05.

## Discussion

Nearly one-third of Minnesota adults who have hypertension without a diabetes diagnosis reported not receiving recommended blood glucose screening in the past 3 years. Adults with hypertension are a group at high risk for developing diabetes; 19.6% and 10.7% of Minnesota adults with hypertension have diagnoses of diabetes and prediabetes, respectively. Because 26% (or approximately 1.1 million) of Minnesota adults report having a hypertension diagnosis (3), approximately 345,000 Minnesota adults with hypertension would report not having recommended blood glucose screening. This is the first state or national assessment of USPSTF diabetes screening rates that also identifies subpopulations less frequently screened. Other state-based analyses of blood glucose screening using BRFSS data described rates in the general adult population or a subset of obese adults that do not align with national guidelines (18–20). Standardized clinical measures for diabetes screening for large population subgroups do not exist and use of All Payer Claims Databases is in its infancy (21). BRFSS provides a unique opportunity to assess statewide trends and monitor the progress of programmatic strategies to improve diabetes screening and hypertension management.

Use of self-reported data adds complexity to data interpretation but does not make the effort less meaningful. Failure to report screening in the past 3 years could reflect not having been screened, lack of awareness that screening was needed or occurred, or both. Lack of awareness could be a function of low health literacy (22,23) and could be used to guide programmatic activity and intervention development.

Consistent with the concept of variable health literacy (22) among adults with hypertension is the association between education and blood glucose screening. Compared with adults with a college degree, adults with less than a college degree were approximately 40% more likely not to have blood glucose testing, as has been observed for people with CHD (16). Higher educational attainment has been associated with greater agreement between self-report of health conditions and the medical record (24).

Health systems factors may influence self-reported screening rates. Not receiving a check-up was associated with lower self-reported screening. Lack of health insurance showed the same relationship in crude models but was not significant after adjustment, likely because of not having had a check-up. In Minnesota, more than 80% of adults with diagnosed hypertension reported visiting a provider in the last year, whereas the US average is 70% (12). Adults who did not use medication to manage their hypertension were less likely to be screened, consistent with results showing that adults who take medication for hypertension or hypercholesterolemia were more aware of their prediabetes status (25). Additional visits for medication management may be required, providing more opportunities for screening; conversely, enhanced screening may reflect clinical assessment of this subgroup being at higher risk.

Variation in screening rates by BMI and age suggest that provider or health systems strategies may also be warranted. After adjustment for other factors, adults younger than 45 years and those with low/normal and overweight BMI were less likely to report screening, as reported previously for adults with CHD (16). The results suggest that providers do not screen all people with hypertension with equal probability, and providers may screen according to guidelines that take age and BMI into account (ie, guidelines from the American Diabetes Association [ADA] or National Institute of Diabetes and Digestive and Kidney Diseases [NIDDK]) (17,26). The results also suggest that awareness of diabetes risk and the need for screening is lower among these groups (ie, adults younger than 45 years and adults with lower BMI) and they are less likely to recall provider-ordered testing. Screening rates among Minnesota adults aged 45 years or older show BMI-related patterns consistent with NIDDK guidelines; in 2011, 63% of overweight or obese adults and 52.1% with low/normal BMI reported screening. NIDDK recommends screening for overweight or obese adults aged 45 years or older and that testing be considered for normal-weight adults of the same age (26). ADA recommends screening all adults aged 45 years or older (17). Given these multiple guidelines, there may be lack of clarity regarding populations eligible for screening.

Strategies addressing barriers related to providers, health systems, and health literacy may improve blood glucose screening rates among adults with hypertension in Minnesota. To address provider awareness and screening, several approaches can be considered: 1) analyzing screening rates using electronic health record data or assessing All-Payer Claims Databases’ provider-ordered screening rates (21), 2) emphasizing that screening adults with hypertension is largely consistent with other screening guidelines, and 3) using clinical decision tools or including blood glucose screening in hypertension management plans. Recent policy changes also may improve access to care. The Affordable Care Act expands the number of people insured and provides an annual preventive visit that could address blood glucose screening at no cost to the patient. Efforts to address health literacy could include awareness campaigns that emphasize the need for blood glucose screening for all adults with hypertension and stress that preventive visits, now a covered benefit under the Affordable Care Act, are opportunities for recommended screening.

Although our analysis is meant to inform programmatic efforts in Minnesota, our findings may be useful more broadly. National-level analysis of diabetes screening rates among adults with CHD found similar rates and screening patterns. Blood glucose screening rates from the 16 states with available 2011 BRFSS blood glucose screening data for all adults with hypertension ranged from 61.9% to 74.1% . Minnesota rates fall midrange, failing to provide evidence that our rates would be highly divergent from results in other states. Univariate analysis of blood glucose screening rates for all adults in Montana (BRFSS 2009) and New York (BRFSS 2008–2009) demonstrated lower screening rates with lower BMI, lower age, and lower educational attainment, consistent with our findings (18,19).

Our study has 5 key limitations. First, we were unable to identify asymptomatic adults with hypertension, the population to which the USPSTF recommendation applies (9), because BRFSS lacks questions about signs and symptoms of diabetes among those without a diagnosis. Second, self-reported measures like diagnosed hypertension are a combination of testing and diagnosis rates and awareness of the diagnosis. Third, the reliability of hypertension and diabetes diagnoses and blood glucose screening is not well-defined. Earlier work suggests high sensitivity for hypertension, moderate sensitivity for diabetes, and high specificity for both (27) (κ, range, 0.7–0.8, for both) (24). Reliability is important from a clinical perspective but becomes less so when addressing issues of patient awareness and health literacy. Fourth, small numbers limited our ability to examine patterns of screening among adults of nonwhite, non-Hispanic race/ethnicity. Finally, we analyzed only 1 year of data. It is unlikely that using a single year of data misrepresents larger trends, given the similarity between 2011 BRFSS estimates in Minnesota and other states. Also, the 19.6% prevalence rate for diabetes among Minnesota adults with hypertension is similar to the rate of 18% to 18.5% reported in 2005–2010 NHANES (28).

Nearly one-third of Minnesota adults with hypertension without diabetes did not report having had blood glucose testing in the past 3 years, as recommended by the USPSTF. Failure to screen and failure to understand the importance of screening may mean missed opportunities for 1) early detection and good clinical management of diabetes and 2) identification of adults with prediabetes and referral to evidence-based interventions that delay or prevent the onset of diabetes (5,6,29) and that may confer additional cardiovascular benefit (30–32). Efforts to improve screening rates should consider a multifaceted approach, addressing provider-, health systems–, and health literacy-related barriers suggested by surveillance data.
